# Genetic and oceanographic tools reveal high population connectivity and diversity in the endangered pen shell *Pinna nobilis*

**DOI:** 10.1038/s41598-018-23004-2

**Published:** 2018-03-19

**Authors:** Marlene Wesselmann, Mercedes González-Wangüemert, Ester A. Serrão, Aschwin H. Engelen, Lionel Renault, José R. García-March, Carlos M. Duarte, Iris E. Hendriks

**Affiliations:** 1 CCMAR, Universidade do Algarve, Gambelas, 8005-139 Faro, Portugal; 2 Global Change Research Group, IMEDEA, Instituto Mediterráneo de Estudios Avanzados, C/Miquel Marqués, 21,07190 Esporles, Spain; 3 LEGOS, Université de Toulouse, IRD, CNRS, CNES, UPS, Toulouse, France; 4 Department of Atmospheric and Oceanic Sciences, University of California, Los Angeles, USA; 5 Instituto de Investigación en Medio ambiente y Ciencia Marina (IMEDMAR-UCV), Universidad Católica de Valencia, C/Explanada del Puerto s/n, Calpe, Alicante, Spain; 6 King Abdullah University of Science and Technology (KAUST), Red Sea Research Center, Thuwal, 23955-6900 Saudi Arabia; 7 Biology department, University of the Balearic Islands (UIB), crta. Valldemossa km 7,5, 07122, Palma de Mallorca, Spain

## Abstract

For marine meta-populations with source-sink dynamics knowledge about genetic connectivity is important to conserve biodiversity and design marine protected areas (MPAs). We evaluate connectivity of a Mediterranean sessile species, *Pinna nobilis*. To address a large geographical scale, partial sequences of cytochrome oxidase I (COI, 590 bp) were used to evaluate phylogeographical patterns in the Western Mediterranean, and in the whole basin using overlapping sequences from the literature (243 bp). Additionally, we combined (1) larval trajectories based on oceanographic currents and early life-history traits and (2) 10 highly polymorphic microsatellite loci collected in the Western Mediterranean. COI results provided evidence for high diversity and low inter-population differentiation. Microsatellite genotypes showed increasing genetic differentiation with oceanographic transport time (isolation by oceanographic distance (IBD) set by marine currents). Genetic differentiation was detected between Banyuls and Murcia and between Murcia and Mallorca. However, no genetic break was detected between the Balearic populations and the mainland. Migration rates together with numerical Lagrangian simulations showed that (i) the Ebro Delta is a larval source for the Balearic populations (ii) Alicante is a sink population, accumulating allelic diversity from nearby populations. The inferred connectivity can be applied in the development of MPA networks in the Western Mediterranean.

## Introduction

Understanding marine connectivity is crucial to implement effective conservation measures and designing representative MPA networks^1^. For most benthic marine species, the exchange of individuals between populations occurs primarily during a pelagic stage^2^. Exchange is influenced by oceanographic processes (currents, winds, fronts or eddies^1^), topography, larval behaviour and species life-history traits^3^. The measurement of connectivity remains extremely challenging due to poor understanding of the interactions between dispersal and oceanic features. However, the combination of oceanographic and genetic tools^4^ has made it possible to estimate the influence of seascape variables on connectivity estimated with genetic markers (e.g.^5–7^,).

The Mediterranean Sea is a global marine biodiversity hotspot where high levels of endemism coexist with intensive anthropogenic pressures on marine biodiversity^8^. This study aims to investigate connectivity patterns in this region as baseline knowledge to enable better design of MPA networks. We use the pen shell *Pinna nobilis* as a model species, a Mediterranean endemic and endangered bivalve. The pen shell can reach more than 1 m length^9^ and is ecologically important in providing hard substratum for colonization by many benthic species in its soft natural habitat, *Posidonia oceanica* meadows^10^. Consequently, any population disruption could have cascading effects on the associated benthic community^11^. Due to anthropogenic impacts (e.g.^12^) and the loss and fragmentation of its natural habitat^13^, the pen shell *P. nobilis* is on the list of endangered species of the Mediterranean Sea (Barcelona Convention, protocol ASPIM Annex 2) and it is protected under the European Council Directive 92/43/EEC (Annex 4). However, despite more than 20 years of protection, abundances and densities of *P. nobilis* are still very low in many areas^14^. Such endangered species with small (effective) population size may be predicted to have low genetic diversity due to bottlenecks and drift, and to have reduced connectivity due to limited fertilization success of propagules when populations have small sample sizes and low density^15^. Thus, assessing diversity and connectivity among *Pinna nobilis* populations is fundamental to help in the design of a network of MPAs for conservation in the Mediterranean Sea.

Until now, the genetic structure of the pen shell has only been studied in the Eastern Mediterranean and in the Tyrrhenian and Sardinian Seas using mtDNA^16–18^ and nuclear^19^ DNA sequences (18S and 28S). Recently, 10 microsatellite loci for *P. nobilis* were designed and tested in two populations from the Balearic Islands^20^. These are faster evolving genetic markers providing higher resolution than mtDNA, rendering them a better choice for studies of contemporary population connectivity and enabling the capture of more recent demographic patterns^21^.

Here, we extended the sampling design of the previous mitochondrial analyses (COI) to the north-Western Mediterranean basin (a region from which genetic information is lacking) in order to understand the genetic variability of *P. nobilis* at a broader scale, improving thus the coverage of the current geographic range. We then zoom into our study area, where we employed a multidisciplinary approach combining microsatellite loci^20^ and Lagrangian simulations of theoretical dispersal probabilities of the larvae based on hydrodynamics and early life-history traits of the species. Specifically, we aim to (i) study the phylogeographical pattern of *P. nobilis* in the Mediterranean with mtDNA markers (COI); (ii) assess the genetic diversity of the populations of *P. nobilis* in the Western Mediterranean Sea using microsatellites; (iii) infer the connectivity pattern between the populations of *P. nobilis* from the Balearic Islands and the Mediterranean mainland Coast of Spain and France; (iv) identify source and sink populations for this species in the Western Mediterranean Sea; and (v) use this information to suggest areas of conservation priority for a network of marine protected areas.

## Material and Methods

### Particle backtracking dispersal model

**Figure 1:**
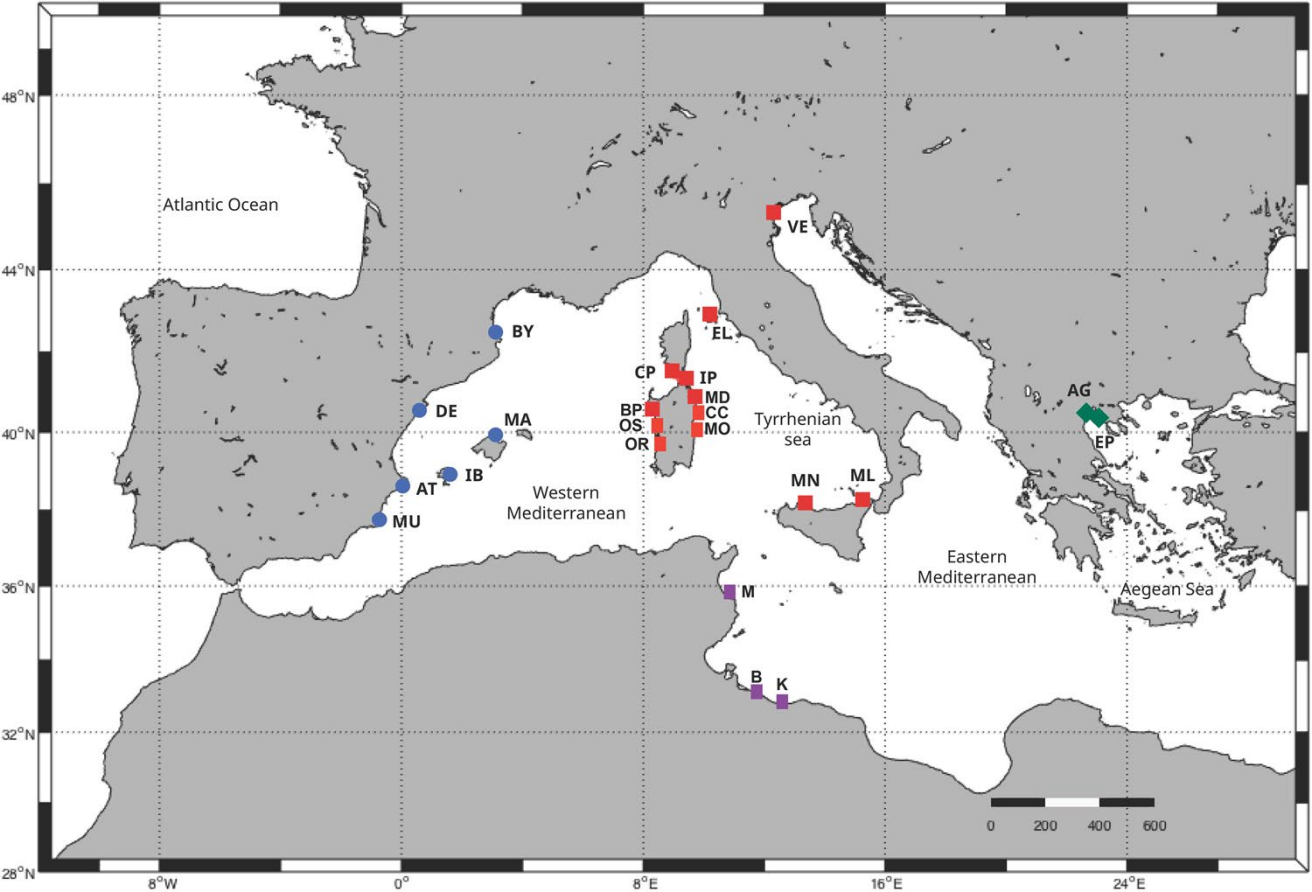
Sampling locations of *P. nobilis*. Present study (blue circles): Banyuls (BY), the Ebro Delta (DE), Alicante (AT), Murcia (MU), Ibiza (IB), Mallorca (MA). Geographic location of data from Sanna *et al*.^18^ (red square): Elba (EL), Cala Pesciu Cane (CP) and Isola Piana (IP) in Corsica, Baia di Porto Conte (BP), Ospedale Marino (OS), Molara (MO), Capo Ceraso (CC), Oristano (OR) and Isola di La Maddalena (MD) in Sardinia, Mondello (MN), Milazzo (ML) and Origina di Siracusa (OR) in Sicily, the Venetian Lagoon (VE). Geographic location of data from Rabaoui *et al*.^17^ (purple square): Monastir (M), El Bibane (B) and El Ketef (K). Geographic location of data from Katsares *et al*.^16^ (green squares): Aegean Sea: Aggeloyesori (AG) and Epanomoi (EP). Map created using QGIS software version 2.18.13.

The oceanic simulations were performed at 6 localities in the Western Mediterranean (Fig. 1), using the UCLA version^22^ of the Regional Oceanic Modeling System (ROMS). ROMS is implemented over a domain that encompasses the Western Mediterranean Sea, from 7.8°W to 9.2°E and from 33.4°N to 44.5°N. The horizontal grid is 681 × 574 points with a resolution of 2.1 km (1/40°) and the vertical discretization considers 42 sigma levels, allowing the representation of mesoscale structures in this region. The bathymetry was acquired from 1′ topography database^23^. The simulations were nested and initialized from the Mercator PSY2V4R3 simulation^24^. Hourly outputs from a regional configuration of the atmospheric model Weather Research and Forecasting (WRF^25^); are used to force ROMS using a bulk formula^26^. Similar configuration has been used and validated by^27^.

Due to great inter annual changes in current patterns the simulations were performed from year 2010–2014. The model was started on June 1^th^ of each year and lasted for a period of 4 months. However, due to the lack of data assimilation in the ocean model, a short spin-up (3 weeks) was used to avoid too much drift of the ocean model from observed conditions. Because of this, simulations were used only from the 23^rd^ of June of each year; corresponding to the beginning of time frame larval propagules are expected in the water column (pelagic period of *P. nobilis* larvae from end June–September).

To assess potential dispersal patterns in the study area, Lagrangian particles (representing the *P. nobilis* larvae) were released and backtracked in the 6 sampling locations (Banyuls, the Ebro Delta, Alicante, Murcia, Ibiza and Mallorca; Fig. 1) during the 5 years selected (2010; 2011; 2012; 2013; 2014). Square networks of 16 particles spaced by 0.03° (1.2–1.6 km) were released daily within the mixed layer at adult sampling sites during the approximate spawning period of *P. nobilis*.

In total, almost ~100,000 particles were launched. Once released, the particles acted as drifters embedded in the circulation flow fields and were backtracked for the pelagic period of *P. nobilis* larvae (end June–September). The particles’ location at each time was determined using a fourth-order *Runge*-*Kutta* method. Thus, the model is able to determine the origin of larvae ending up at each sampling location, identifying potential source populations.

### Sampling

To investigate the genetic structure of *P. nobilis*, tissue samples from 6 localities (134 individuals in total; sample sizes detailed in Table 1) in the Western Mediterranean Sea (Fig. 1). Samples were obtained by SCUBA diving during the summer of 2014, in the location of Banyuls (beside the Marine Reserve of Cerbère-Banyuls), the Ebro Delta (within the semi-enclosed Alfacs Bay), Alicante (in the bay of Calpe) and Murcia (within the semi-enclosed bay of Mar Menor). The samples collected in Mallorca (Pollença, in the North) and Ibiza (in the South) were obtained around the Balearic Islands during the summer of 2011. The sampling method was specific for *P. nobilis*, preventing killing: valves of each fan mussel were maintained open with a special aluminium clip, while a Hartman alligator forceps was used to take a small portion of mantle tissue (20 mg approx.). This opening device was designed to obtain a maximum aperture of 1.2 cm, according to the natural range of shell aperture of *P. nobilis*^9^. The mantle tissue of individuals was preserved in 95% ethanol and stored in 1.5 ml eppendorf tubes.

**Table 1:** Genetic diversity estimates based on 10 microsatellite markers and Cytochrome Oxidase I (597 bp) of adults and juveniles of *P. nobilis* populations from the Western Mediterranean: Banyuls (BY), the Ebro Delta (DE), Alicante (AT), Murcia (MU), Ibiza (IB), Mallorca (MA). *N* number of individuals, *A* allelic richness, *PA* private alleles, *H*_*O*_ observed heterozygosity, *H*_*E*_ expected heterozygosity, *F*_*IS*_ is the imbreeding coeficient, *Hap* n° of haplotypes and in brackets n° of singletons, *Ps* n° of polymorphic sites, *H* haplotype diversity and Π nucleotide diversity. *F*_*IS*_ values with * are significant at *P* < 0.05.

	Site	Microsatellites						Mitochondrial DNA (COI)				
		*N*	*A*	*PA*	*H*_*O*_	*H*_*E*_	*F*_*IS*_	N	Hap	Ps	H	Π
Adults	BY	20	8.4	5	0.560	0.707	0.152*	9	5(3)	7	0.8889	0.0035
	DE	22	8.7	4	0.610	0.719	0.127*	9	3(2)	3	0.4167	0.0007
	AT	20	10	6	0.595	0.777	0.226*	10	7(5)	13	0.8667	0.0062
	MU	22	8.9	7	0.533	0.738	0.269*	9	2(0)	1	0.5000	0.0008
	IB	25	9	4	0.540	0.732	0.249*	10	6(3)	5	0.7778	0.0016
	MA	25	9.4	8	0.566	0.732	0.233*	10	7(3)	8	0.9111	0.0032
Juveniles	BY	35	10.6	—	0.579	0.737	0.217*	—	—	—	—	—
	MA	35	10.6	—	0.603	0.752	0.199*	—	—	—	—	—

In order to assign juveniles to adult *P. nobilis* populations, juveniles were sampled with larval collectors modified from^28^. Two collectors per sampling locality were deployed in June and recollected in November 2014. Each device consisted of 2 independent replicate polyethylene mesh bags for oyster aquaculture filled with a mesh (13 m-long and 0.1-m-wide, and mesh width of 0.005 × 0.005 m) that mimic *P. oceanica* rhizomes. These bags were attached at 5 m depth to a rope that was fixed to a buoy. The buoy was suspended at 2–3 m depth to keep the bags afloat. After recollection, 90 post larvae were obtained in Banyuls, 35 in Mallorca and 1 in Alicante. These recruits were stored in 1.5 ml eppendorfs with 95% ethanol. No post larvae were obtained in the other localities, as collectors were lost due to unknown reasons (storms or human contact). In order to have an equivalent and representative number of post larvae of each location, solely 35 post larvae from Banyuls and Mallorca were used.

### Molecular analysis

#### Mitochondrial markers: COI

DNA was extracted from 57 individuals, around 10 per site, following the protocol of^29^. A fragment of 590 bp of the cytochrome oxidase I gene was amplified by PCR using the universal primers HCO2198 and LCO1490^30^. Each PCR reaction mixture was performed in 20 μl total volume containing 50 ng of 1:50 diluted genomic DNA, 1.4 μl MgCl_2_ (25 mM), 0.16 μl dNTPs (2 mM), 3 μl of each primer (10 μM), 2 μl PCR GoTaq Flexi buffer and 0.1 μl GoTaq Flexi DNA Polymerase (Promega). Amplifications were performed in a thermocycler (Applied Biosystems®): 2 min at 95 °C, 35 cycles at 95 °C for 50 s, 50 s at an annealing temperature of 49 °C, and, 1 min 20 s at 70 °C and a final extension at 72 °C for 7 min. Amplicons were sequenced in an automated capillary sequencer (ABI PRISM 3130).

#### Microsatellite markers

Using the same DNA extraction protocol mentioned above, genomic DNA was extracted from 204 individuals (134 adults and 70 post-larvae). Afterwards, all 204 individuals were genotyped using ten microsatellite loci^20^.

Microsatellites amplification was carried out in different PCR reactions (each msat was a stand alone PCR) and were composed of 20 μl of reaction mixture, containing 50 ng of 1:50 diluted DNA template, 1.2–1.6 μl of MgCl_2_ (25 mM), 1 μl dNTP’s (2 mM), 0.5 μl mM of each primer (10 μM) (the forward was labelled with FAM, HEX or NED), 4 μl GoTaq Flexi buffer and 0.1 μl GoTaq Flexi DNA Polymerase (Promega). PCR conditions were as follows: 3 min at 95°, 35 cycles of 50 s at 95°, 50 s at annealing temperature^20^, 1 min at 72° and a final elongation for 5 min at 72 °C. Amplicons were separated using an ABI PRISM 3130 automated capillary sequencer. Alleles were scored in STRAND software v. 2.4.59^31^ using the 350 ROX^TM^ size.

### Data analysis

#### Mitochondrial DNA (Cytochrome oxidase I)

The 57 sequences amplified in the present study from 6 different sites of the Western Mediterranean (Banyuls, the Ebro Delta, Alicante, Murcia, Ibiza and Mallorca; Fig. 1) were aligned using Bioedit Sequence Alignment Editor v. 7.2.5^32^. After adding 235 COI sequences available from the literature from 24 *Pinna nobilis* in the Tyrrhenian and Sardinian Seas and in the Eastern Mediterranean Sea (16, 17, 18; Fig. 1), we constructed two different datasets. The first one included 57 COI sequences of 590 bp obtained in the present study from the Western Mediterranean populations. The second one included 292 COI sequences of 243 bp (maximum overlap between sequences of the different studies previously published) from all over the Mediterranean (Table S1). Only localities with a minimum number of 9 individuals sequenced available in the literature^16–18^ were chosen for this second analysis. Haplotype and nucleotide diversity and pairwise *F*_*ST*_ values were calculated using ARLEQUIN v. 3.11 software^33^. The same program was used to perform Tajima’s D^34^ and Fu’s F_s_^35^ neutrality test and mismatch distribution analyses^36^ and the Ramos-Onsins and Rozas’ R2 test^37^ was performed with DNASP 5.10.01^38^. Significance was tested by using 10,000 random permutations. A statistical parsimony network of haplotypes was built with TCS software v. 1.21^39^.

#### Microsatellites

Allelic frequencies, observed (*H*_*O*_) and expected (*H*_*E*_) heterozygosities were estimated using ARLEQUIN v. 3.11^33^. Deviations from Hardy-Weinberg equilibrium (HWE) were characterized by the inbreeding coefficient (*F*_*IS*_) with GENETIX v. 4.05^40^ and P-values where calculated GENEPOP v. 4.2^41^. Putative scoring errors were checked with MICRO-CHECKER v. 2.2.3^42^ and FreeNA v. 9.3^43^ was used to to evaluate possible differences between estimates of global and pairwise *F*_*ST*_ values including and excluding null alleles (ENA method for estimating *F*_*ST*_ values). The assumption of linkage equilibrium was tested in GENEPOP 4.2^42^.

The quantification of genetic differentiation was established through *F*_*ST*_^44^ with FreeNA v. 9.3^33^. However, due to recent concerns raised about the usage of *FST* in highly polymorphic systems, we also calculated *D*_*ST*_^45^. Pairwise *DST* statistic estimates and their significance using 1,000 bootstrap replicates were obtained using the DEMETICS^46^ package in R software^47^. In addition, genetic differences were further analysed using a correspondence analysis (CA) of the allele frequencies to detect differences and similarities between populations, using the BiodiversityR^48^ package in R software. Finally, the population genetic structure was explored with the software STRUCTURE v. 2.3^49^, a Bayesian clustering method that assigns individuals to groups (k) without prior information. The analysis was run under the admixture model^49^ with K ranging from 1 to 9 (n° of sampled populations plus three) and 20 independent runs with a burn-in of 100,000 iterations and a run-length of 500,000 iterations. The most likely K (number of groups) was inferred using the DeltaK criterion^50^ with STRUCTURE HARVESTER^51^.

Juveniles were assigned to adult populations using GENECLASS v. 2.0^52^ under the Bayesian assignment method^53^. Adult genotypes of each of the six sites were used as reference populations and juveniles (obtained solely in Mallorca and Banyuls) were then either assigned or excluded from each of the populations using the Monte Carlo re-sampling approach (n: 10,000)^54^. Juveniles were considered immigrants when the probability of been assigned to any population was lower than 0.05 (type I error). When a juvenile showed probabilities of assignment greater than 0.05 to only one population it was assigned to that population. Finally, when a juvenile was assigned to more than one population (with p > 0.5) it was considered to have originated from an unsampled source.

Estimates of migration rates (*M* = m/μ, where *m* is the fraction of the new immigrants in the population per generation and μ is the mutation rate of the gene) were obtained with the program MIGRATE v. 3.2.7^55^. In order to determine source and sink populations between connected localities and to obtain more accurate results (Migrate-n works better with ≤3 populations), we divided our localities in 3 analysis according to the clusters identified by STRUCTURE. In each analysis we included the three sites that showed the highest estimated membership proportion to each of the clusters: (1) BA, DE and AT, (2) AT, MU and IB and (3) DE, IB and MA. These analyses were conducted under the Bayesian inference approach and involved a Markov Chain Monte Carlo search of 10000 discarded trees per chain followed by 5000 steps with parameters recorded every 500 steps, a uniform prior on theta (Min:0.0, Max:50.0, Delta:1.0) and a uniform prior on migration (Min:0.0, Max:50.0, Delta:1.0). Convergence was checked by inspecting posterior distributions of the parameters.

Finally, to determine whether genetic differences were related to the Median Oceanographic Distance (MOD) and the Median Oceanographic transport Time of larvae (MOT), Mantel tests were implemented (10,000 permutations^56^); in IBD v. 1.52. The oceanographic distance (MOD) and time (MOT) between each grid cell of the Lagrangian trajectories was quantified as in^57^:

1 $$MOD(i,j)=\frac{1}{M}\sum _{n=1}^{n=M}{D}_{n}(i,j)$$

2 $$MOT(i,j)=\frac{1}{M}\sum _{n=1}^{n=M}{T}_{n}(i,j)$$

where D(*i*, *j*) is the distance from grid cell *i* to grid cell *j*, *M* is the number of particle transitioning from *i* to *j* and T(*i*, *j*) is the transit time from grid cell *i* to grid cell j.

For the trajectory of each pair of population, all intermediate positions were used to calculate the MOD and MOT. The shortest average distance (MOD) and time (MOT) for each pair of populations was used as in^6^. Unfortunately, the MOD and MOT of several pairwise sites were not possible to compute, as not all grid cells were connected (e.g. Banyuls and Murcia). In those cases, the MOD and MOT of that pairs of population was established to be ≥than the maximum oceanographic distance and time calculated for all the populations.

## Results

### Dispersal models: Lagrangian simulations

The numerical Lagrangian trajectories did not show larvae exchange between Banyuls and the rest of localities and between Murcia and the Ebro Delta and Mallorca. This lack of connection might be related to the long oceanographic distances and transport time between these sites. Actually, the minimum Oceanographic Transport Time between these sites (35 days; Table S2), exceeded the maximum pelagic larvae time of *P. nobilis* (20 days: Iris Hendriks pers. comm.). The rest of the pairs of populations exchanged larvae between each other within their pelagic life-time (Minimum oceanographic transport time between sites was ≤20 days, with the exception of Alicante-Mallorca; Table S2).

**Figure 2:**
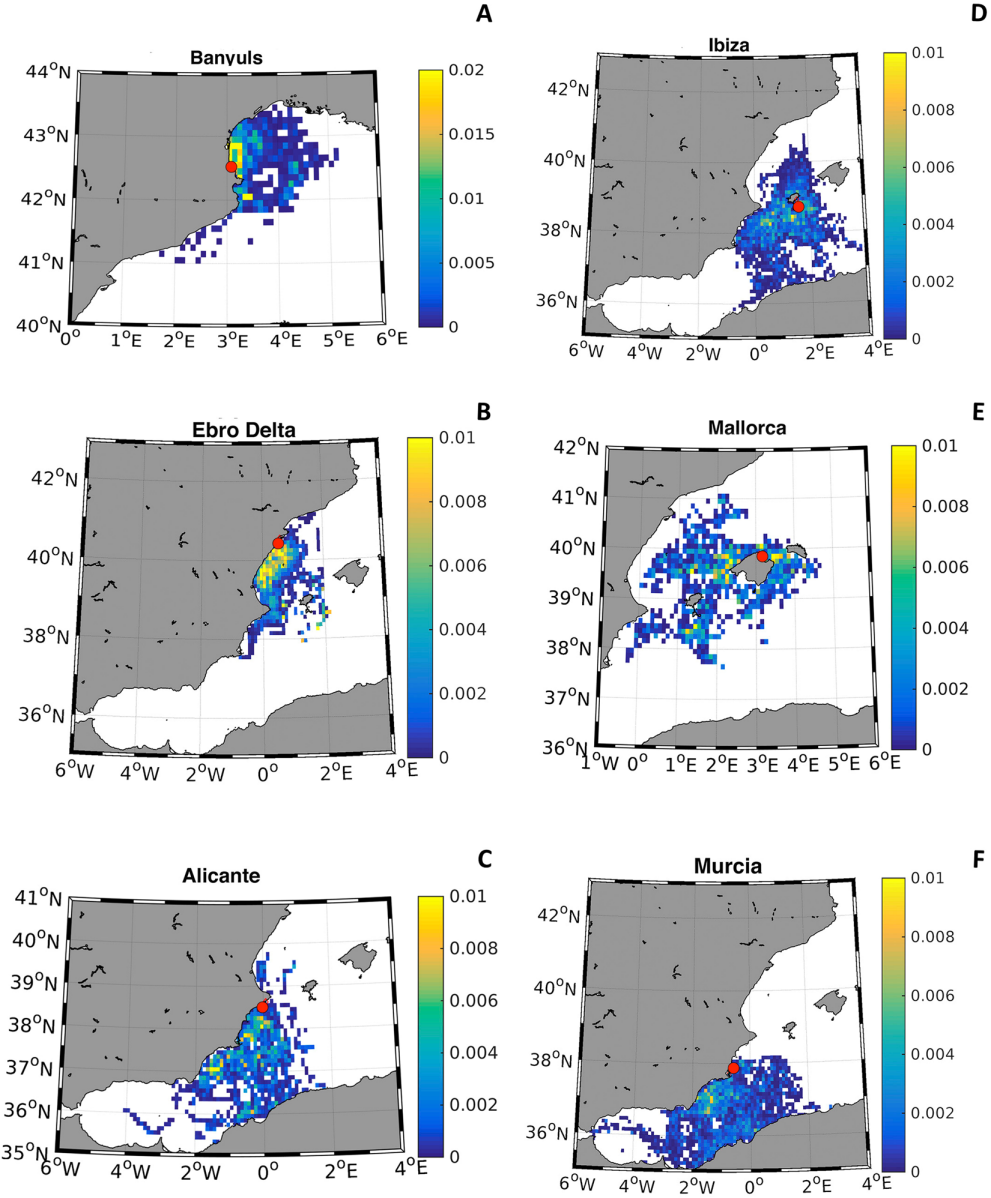
Map showing the potential dispersion capacity of *P. nobilis* larvae during their maximum pelagic larvae time (20 days) based on a hydrodynamic numerical simulations (ROMS) with seeding points at sampled adult populations (**A**): Banyuls, (**B**): the Ebro Delta, (**C**) Alicante, (**D**) Ibiza, (**E**) Mallorca, (**F**) Murcia from 2010 to 2014. Colours show the Probability Density Function (PDF) of the particle-larvae to end up in the adult population (marked with a red circle). Map generated with Matlab R2014b (https://www.mathworks.com/) using data from ROMS.

In general, according to the hydrodynamic model (Fig. 2A–F), our study sites were influenced mainly by the Northern Current (NC), Algerian Current (AC) and Balearic Current (BC). Most of the larvae arriving to Banyuls were greatly influenced by the NC as they are expected to come from north of this population (from Banyuls until the “Park Naturel Régional de la Narbonnaise”; not sampled in this study), except some larvae coming from Cap de Creus and Medes Islands (Fig. 2A). On the contrary, the NC is not expected to influence the larvae arriving to the Ebro Delta, as the back-tracking model predicted that the larvae arriving there were originated from the coast of the province of Castellon and Columbretes Islands (not sampled in this study) and with a lower probability from Calpe (Alicante) (Fig. 2B). The model indicates that the AC disperses larvae directly from the coast of the province of Murcia to Alicante (Fig. 2C). In the simulation, the majority of *P. nobilis* larvae from Ibiza come from the South of Ibiza and Formentera, although some may also originate from Alicante and Murcia following the retroflection of the NC into the BC along the North Western slope of the Archipelago (Fig. 2D). Larvae of the North of Mallorca (Pollença), seem to come with more probability from other parts of the Island and in some occasions from Ibiza and Alicante (Fig. 2E). Finally, he larvae arriving to Murcia were also influenced by the AC, originating to a greater extent from south of this locality and to a lesser extent from the African northern coast (Fig. 2F).

### Mitochondrial DNA

#### Genetic diversity

The 590 base pair (bp) fragment of COI was aligned with available Genbank sequences, obtaining a 243 bp sequence alignment for 292 *P. nobilis* individuals from 24 sites in the Mediterranean, defined by 32 polymorphic sites comprising a total of 43 haplotypes (Table S3). Most haplotypes (61%) were found only at a single locality, as 17 were shared haplotypes and 23 were singletons. The highest values of haplotype and nucleotide diversity were found in the populations of: Alicante, Elba Island, Corsica (Isola plana), the Venetian Lagoon and Sicily (Milazzo). The lowest values of haplotype and nucleotide diversity were found in Murcia, the Ebro Delta, Aggeloyesori in the Aegean Sea and el Ketef in the Tunisian population (Table S3). The haplotype sequences were registered in Genbank (accession n°: KY321755-KY321811).

At a Western Mediterranean scale, the 590 base pair (bp) of COI also showed low nucleotide and high haplotype diversity across the 57 individuals collected at six localities (Table 1). Sequences revealed 69 polymorphic sites and a total of 21 haplotypes of which 5 were shared haplotypes and 16 were singleton haplotypes. The highest number of haplotypes and haplotype diversity were found in Alicante and Mallorca, whereas the lowest occurred in the Ebro Delta and Murcia (Table 1).

#### Population structure

The Mediterranean-wide analysis (using 243 bp of COI) showed no clear division between Western and Eastern Mediterranean basins and indicated the Venetian Lagoon as the most distinct population. At a Western Mediterranean scale, genetic similarities were found between Delta, Alicante, Mallorca, Ibiza and the Western Mediterranean haplogroup defined by (18: Elba Island, Sardinia, Corciga and Sicily). In contrast, Banyuls and Murcia showed significant genetic differentiation with this haplogroup (Table S4). The Mediterranean-wide analysis (using 243 bp of COI) as well as longer COI sequences of the Western Mediterranean (using 597 bp of COI) did not detect genetic differentiation between the sampled populations of the present study (Banyuls, The Ebro Delta, Alicante, Murcia, Ibiza and Mallorca) (Table S5).

#### Demographic inference

**Figure 3:**
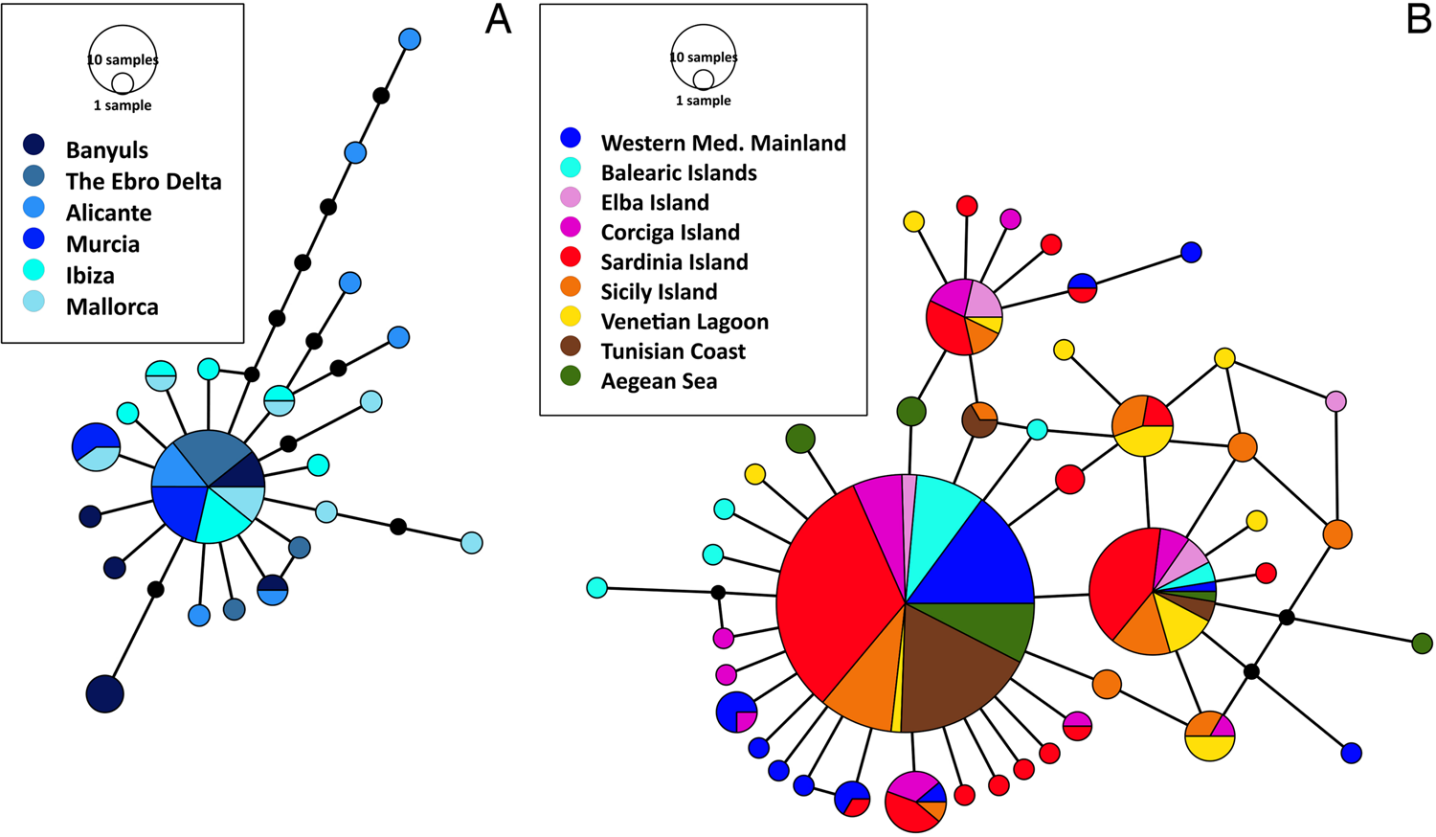
Statistical parsimony network based on COI sequence haplotypes of *Pinna nobilis* for the Western Mediterranean (**A**) and the entire Mediterranean basin (**B**). The circles represent haplotypes and size of each of them is proportional to haplotype frequency. Connection lines between circles represent mutations and black dots corresponding to mutational steps. Map generated with PopArt 1.7.

Considering all Mediterranean localities (243 bp) and the sampled Western Mediterranean localities with longer COI sequences (597 bp) the statistical parsimony network of the COI haplotypes exhibited a star structure (Fig. 3). The two most common haplotypes (1b and 2b) were shared among all the Mediterranean populations, comprising 55% and 13% respectively of all the sampled individuals across the entire study region. The third most common haplotype^3b^ was restricted to populations from the Tyrhenian and Sardinian Sea (Elba, Sardinia, Corsica, Sicily and the Venetian Lagoon), which shared in total six haplotypes (1b, 2b, 4b, 5b, 6b & 7b) with populations from the Western Mediterranean mainland. Longer COI sequences revealed solely 5 shared haplotypes: 1a (present in 50% of the individuals), 2a (shared between Mallorca and Murcia), 3a (shared between Banyuls and Alicante) and two haplotypes shared between Mallorca and Ibiza (4a and 5a). All locations, except Murcia, presented exclusive haplotypes (Table 1). The majority of the singletons occupied a distal position in the network, an indication of more recent haplotypes in accordance with the criteria of^58^.

The mismatch analysis of both analyses of COI, 1) 24 populations from the whole Mediterranean basin; 2) 6 Western Mediterranean populations, revealed an unimodal distribution (^59,60^; Fig. S1a & S1b). The dataset exhibited negative and significant Tajima’s D and Fu’s FS values, suggesting a sudden population expansion in the Ebro Delta, Ibiza and Mallorca (Table S6 and S7). However, the Ramos-Onsins’ R2 test, which is more powerful than *D* and *F*_*ST*_ at small sample sizes, only corroborated this sudden population expansion in Ibiza and Mallorca (Table S6) and also detected it in the populations of Alicante and Aggeloyesori (Table S6 and S7).

### Microsatellites

#### Genetic diversity

Mean allelic diversity (*A*) and mean expected heterozygosity (*H*_*E*_) of *P. nobilis* adults were high (*A*: 9.2, *H*_*E*_ = 0.740), with Alicante showing the highest and Banyuls the lowest values. *P. nobilis* juveniles (only obtained in sufficient numbers at two locations, Mallorca and Banyuls) presented similar allelic diversity and expected heterozygosity values (Table 1).

The inbreeding coefficient (*F*_*IS*_) was positive and significant at all locations (Adults and Juveniles). None of the loci presented large allele dropout or any other evidence of genotyping errors; however, evidence of null alleles was detected. Although the high *F*_*IS*_ observed could be explained by the presence of null alleles at some loci, these were not removed from the analyses as no bias due to null alleles was found when comparing the original data set with that excluding null alleles (Table S8).

#### Population genetic Structure

The pairwise *F*_*ST*_ estimates did not detect population differentiation, although *D*_*ST*_ estimates showed significant genetic differentiation between Banyuls and Murcia and between Mallorca and Murcia (Table 2). The correspondence analysis based on the allele frequencies (58.6% of the total variance for the two first ordination axes) revealed a weak geographic structure showing three major groups: (1) Banyuls on the positive part of the axis I; the axis II separated (2) The Ebro Delta and the Balearic localities (Ibiza and Mallorca) on the positive site and (3) Alicante and Murcia on the negative site (Fig. S2). The STRUCTURE analysis, using the^48^ method for estimating the true K, indicated also that there are three clusters in the data set. Banyuls exhibited a higher likelihood of belonging to cluster 1, Mallorca to cluster 2 and Murcia to cluster 3. By contrast, the individuals from the Ebro Delta, Alicante and Ibiza were not assigned predominantly to either one of these three clusters, showing a strong ad-mixture (Table 3).

**Table 2:** Estimates of pairwise microsatellite *F*_*ST*_ values (below diagonal) and *D*_*ST*_ (above diagonal) among population of *P. nobilis* from the Western Mediterranean: Banyuls (BY), the Ebro Delta (DE), Alicante (AT), Murcia (MU), Ibiza (IB), Mallorca (MA). *F*_*ST*_ and *D*_*ST*_ values with * are significant after Bonferroni correction (P < 0.003).

Locations	BY	DE	AT	MU	IB	MA
Banyuls	—	0.0870	0.0999	0.1183*	0.1018	0.0847
Ebro Delta	0.0120	—	0.0318	0.0176	0.0112	0.0397
Alicante	0.0088	0.0010	—	0.0247	0.0584	0.0460
Murcia	0.0180	0.0023	−0.0059	—	0.0172	0.1168*
Ibiza	0.0167	−0.0037	0.0056	−0.0036	—	0.0482
Mallorca	0.0166	0.0024	0.0022	0.0120	−0.0016	—

**Table 3:** Estimated membership proportion in each of the three genetically differentiated clusters (k = 3) identified by STRUCTURE.

Locality	Cluster 1	Cluster 2	Cluster 3
Banyuls	0.489	0.205	0.306
Ebro Delta	0.311	0.365	0.324
Alicante	0.380	0.284	0.337
Murcia	0.306	0.224	0.470
Ibiza	0.291	0.350	0.359
Mallorca	0.307	0.474	0.218

No genetic differentiation was found between Juveniles from Banyuls and Mallorca and their respective adult populations (Table S9).

#### Source and sink populations

According to Migrate-n, the Ebro Delta could act as a sink population, receiving genes from Banyuls and Alicante (analysis 1). In contrast, the migration rates of the population from Alicante suggest that these populations could act as a sink population, receiving genes from Murcia (analysis 2). The migration rates of the Ebro Delta showed a predominant gene flow from this locality to Ibiza and from this population to Mallorca, pinpointing the Ebro Delta as a potential source population for the Islands (analysis 3) (Table S10).

Assignment tests demonstrated that only 10% of the sampled juveniles were originated in one of the adult populations (Table S11). Juveniles collected in Mallorca were assigned to DE (1), AT (3), MA (2) and assignment success of juveniles collected in Banyuls was very low; only one juvenile was reassigned to Banyuls. The large majority of sampled juveniles (80%; 29 from Banyuls and 27 from Mallorca) were designated as coming from an unsampled source (p ≤ 0.05). The remaining individuals (10%) were left “unassigned” (Table S11).

**Figure 4:**
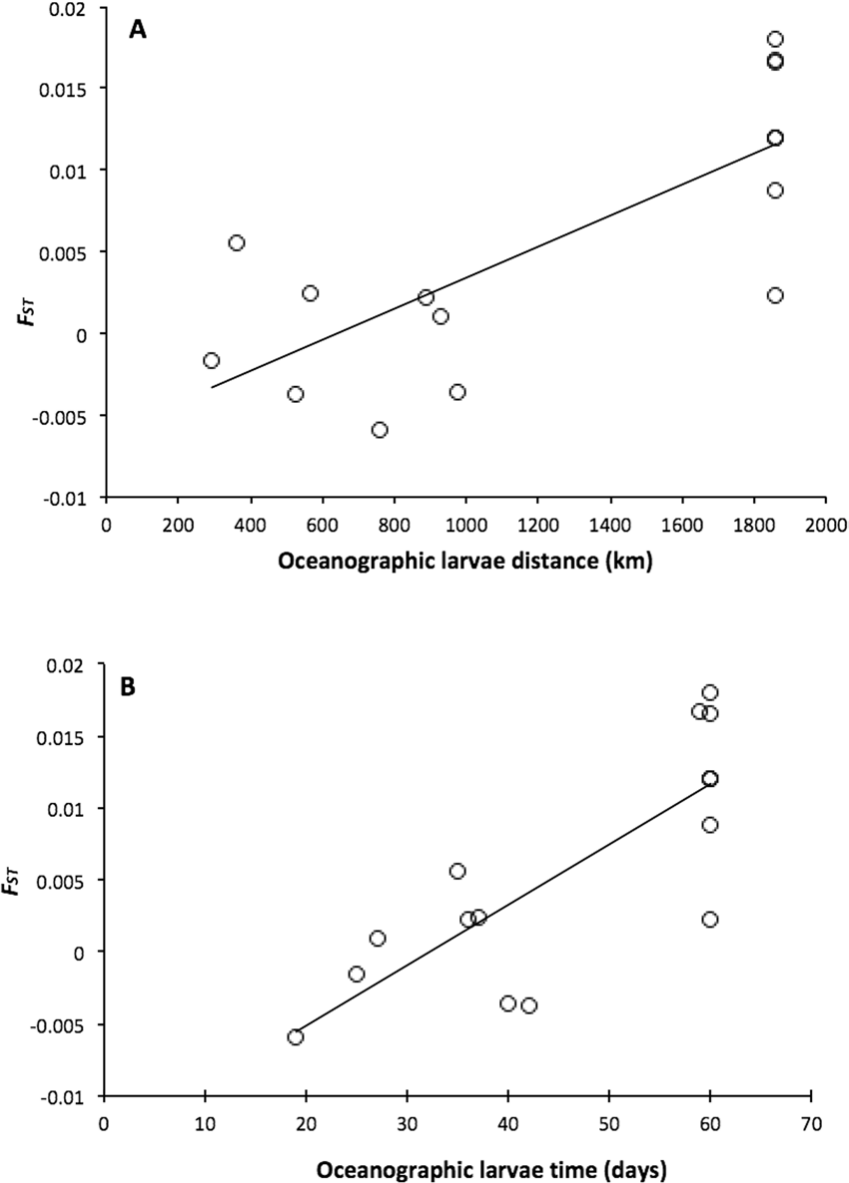
Relationship between pairwise *F*_*ST*_ and Median oceanographic distance (**A**) and Median Oceanographic larvae transport time (**B**) for 6 populations of *P. nobilis* in the Western Mediterranean obtained with microsatellite loci.

Despite the weak genetic differentiation among sites, a significant pattern of isolation-by-distance using *F*_*ST*_ (genetic differentiation estimate) and Median Oceanographic Distance and Median Oceanographic Transport Time was found (Table S2, Fig. 4). The oceanographic transport time was a much better predictor of genetic differentiation than the oceanographic distance; this indicates that the patterns of genetic connectivity are consistent with the backtracking model.

## Discussion

This study shows that on a broad scale, *P. nobilis* populations (sampled along the Western Mediterranean and combined with previously published data of the entire Mediterranean) have high diversity and low inter-population differentiation. This discovery has strong consequences for conservation of this endangered species, as it does not support the highest concern hypothesis of small isolated populations. The results of the multiple analyses all indicate that *P. nobilis* throughout the Mediterranean comprises one lineage that underwent a recent population expansion from a single original population with low effective population size, as hypothesized by^18^. The causes of this past event might have been linked to historical variations in climate and/or habitat availability, given the strong dependence of the species on seagrass habitat.

The populations of *P. nobilis* from the Venetian Lagoon differed from most of the studied *P. nobilis* populations, likely as a result of the semi-enclosed nature of the Adriatic Sea. Genetic differentiation between the Western and Eastern Mediterranean was not detected, possibly due to the low resolution of the analysis (243-bp COI sequences). Although^18^, with slight longer COI sequences (338-bp), detected subdivision between Western and Eastern basins, which, together with the low diversity observed in the eastern side, supports the hypothesis of an eastward expansion during the colonization of the Mediterranean. This has been proposed as the most common route of natural colonization of the Mediterranean in marine species^61^, although there is evidence for other patterns (e.g.^61,62^). A hypothetical eastward expansion would also agree with the suggestion of^63^ that the *Pinnidae* originated and diversified in the Indo-pacific and subsequently invaded the Atlantic Ocean and the Mediterranean Sea eastwards.

Within the Western Mediterranean, the connectivity pattern of *P. nobilis* estimated with higher resolution genetic markers (microsatellites) was strongly influenced by oceanographic currents. This result highlights the importance of ocean currents and pelagic larvae transport time in shaping the population connectivity of *Pinna nobilis* in the study region. The considerable distance between Banyuls-Murcia and Mallorca-Murcia resulted in significant genetic differentiation between these sites, following a pattern of isolation by distance, as described in^64,65^. Banyuls and Mallorca also experienced limited gene exchange, possibly due to the Balearic Front, a density barrier created by the NC flowing southward along the Western Mediterranean Coast and the Atlantic water flowing north-eastwards^66^. This genetic break was also been described for several fish species^67^.

Microsatellite loci and Lagrangian simulations revealed no isolation of the Balearic *P. nobilis* populations with the mainland. The locality of Ibiza appeared to be a key population in order to ensure connectivity, receiving genes from the mainland (the Ebro Delta, Alicante and Murcia) and exporting them to Mallorca. Migration rate estimates pointed the Ebro Delta as a source population, exporting genes to Ibiza and the latter to Mallorca. It is noteworthy that over 90,000 *P. nobilis* individuals inhabit the waters of Alfacs Bay, the largest population so far reported in shallow water^68^. This could be attributed to optimal growth conditions at this locality, because semi-enclosed bays such as Alfacs Bay in the Ebro Delta, with low hydrodynamic energy and a certain input of organic material, favour the growth of *P. nobilis*^69^. If we consider the number of individuals able to spawn in this source population, many sites of the Balearic Islands could benefit from the number of exported larvae. The southern shore of Alfacs Bay (the Banya Sandspit) was included in the Ebro Delta Natural Park in 1986 and is also part of the Natura 2000 site^70^. However, free access to high-density areas of *P. nobilis* and the lack of regulation of the boating activity is causing damage and mortality of individuals in the bay^68^.

Migration patterns reflected the influence of the AC, which enable larvae to be transported from Murcia to Alicante, the latter accumulating genetic diversity as a sink population. Both genetic markers detected the highest genetic diversity in Alicante. Genetic diversity may be high as a consequence of long-term stability of large populations (absence of bottlenecks) but higher diversity might also simply result from a sink population structure. These scenarios do not have the exact same consequences for species conservation, as the second case suggests a population whose high diversity cannot be maintained in the absence of migration, should the sources be impacted.

The connectivity pattern of *P. nobilis* here inferred provides useful information to be applied in the development of a MPA network in the Western Mediterranean. This network of MPAs should protect areas with specific interest in terms of their diversity and connectivity with other populations of *P. nobilis*, as the functioning of populations is dependent upon processes of reproduction and recruitment from a surrounding area^71^. The Ebro Delta should be included in a network of MPAs as a source population for the Balearic Islands. Localities with high genetic diversity, like Alicante (Calpe), should also be included in a network of MPAs, as they retain the genetic diversity of *P. nobilis* in the Western Mediterranean. Finally, this network should maintain connectivity between the Mainland with the Balearic Islands to prevent isolation and avoid the loss of genetic diversity. This could be accomplished by protecting the sampled location of Ibiza, which presents gene flow with the populations from the localities of Alicante, the Ebro Delta and Murcia.

Unfortunately, in the summer of 2016, an haplosporidium like parasite caused a mass mortality event (MME) with mortality rates up to 100% of the *P. nobilis* populations of the Central Spanish Mediterranean sea^72^, among them, Alicante, Murcia, Ibiza and Mallorca. Our study highlighted Alicante as one of the populations with the highest genetic variability values in all the Mediterranean Sea (Tables 1 and S3), which means that this event supposed not only the loss of the densest *P. nobilis* populations^72^ but also one of the most diverse ones. However, CO1 sequences (243 bp) suggest that there are populations with high diversity values in the Tyrhenian and Sardinian seas that share several haplotypes with the Western Mediterranean populations (Fig. 3) that has not been impacted: Elba, Isola Plana in Corsica and Milazzo in Sicily (Table S3). In this context, it would be worth to corroborate this result with faster genetic markers with higher resolution, extending the microsatellite analysis to the whole Mediterranean basin in order to understand if the population impacted from the MME are connected and could be naturally recovered from the *P. nobilis* populations located in the Tyrrhenian and Sardinian Seas.

## Electronic supplementary material

Supplementary Tables

## References

[1] Palumbi SR (2003). Marine reserves and ocean neighborhoods: The spatial scale of marine populations and their management. Annu. Rev. Environ. Resour..

[2] Cowen RK, Sponaugle S (2009). Larval dispersal and marine population connectivity. Annu. Rev. Mar. Sci..

[3] Shanks AL (2009). Pelagic larval duration and dispersal distance revisited. Biol. Bull..

[4] Galindo HM, Olson DB, Palumbi SR (2006). Seascape genetics: a coupled oceanographic-genetic model predicts population structure of Caribbean corals. Curr. Biol..

[5] Alberto F, Raimondi P, Reed D (2010). Habitat continuity and geographic distance predict population genetic differentiation in giant kelp. Mol. Ecol..

[6] Alberto F (2011). Isolation by oceanographic distance explains genetic structure for *Macrocystis pyrifera* in the Santa Barbara Channel. Mol. Ecol..

[7] Sunday JM, Popovic I, Palen WJ, Foreman MGG, Hart MW (2014). Ocean circulation model predicts high genetic structure observed in a long-lived pelagic developer. Mol. Ecol..

[8] Coll M (2010). The Biodiversity of the Mediterranean Sea: Estimates, Patterns, and Threats. PLoS ONE.

[9] García-March JR (2007). Population structure, mortality and growth of *Pinna nobilis* Linnaeus, 1758 (Mollusca, Bivalvia) at different depths in Moraira bay (Alicante, Western Mediterranean). Mar. Biol..

[10] Basso L (2015). The pen shell, *Pinna nobilis*: A review of population status and recommended research priorities in the Mediterranean Sea. Adv. Mar. Biol..

[11] Rabaoui L, Tlig-Zouari S, Cosentino A, Hassine OKB (2009). Associated fauna of the fan shell *Pinna nobilis* (Mollusca: Bivalvia) in the northern and eastern Tunisian coasts. Sci. Mar..

[12] Hendriks IE (2013). Boat anchoring impacts coastal populations of the pen shell, the largest bivalve in the Mediterranean. Biol. Conserv..

[13] Marbà N (2005). Direct evidence of imbalanced seagrass (*Posidonia oceanica*) shoot population dynamics in the Spanish Mediterranean. Estuaries..

[14] Guallart, J., Templado, J. *Pinna nobilis*. In: VV.AA., Bases ecológicas preliminares para la conservación de las especies de interés comunitario en España: Invertebrados. Ministerio deAgricultura, Alimentación y Medio Ambiente. Madrid, pp. 81 (2012).

[15] Deboer T (2008). Phylogeography and limited genetic connectivity in the endangered boring giant clam across the Coral Triangle. Conserv. Biol..

[16] Katsares V, Tsiora A, Galinou-Mitsoudi S, Imsiridou A (2008). Genetic structure of the endangered species *Pinna nobilis* (Mollusca: Bivalvia) inferred from mtDNA sequences. Biologia..

[17] Rabaoui L (2011). Genetic variation among populations of the endangered fan mussel *Pinna nobilis* (Mollusca: Bivalvia) along the Tunisian coastline. Hydrobiologia..

[18] Sanna D (2013). Mitochondrial DNA reveals genetic structuring of *Pinna nobilis* across the Mediterranean Sea. PLoS ONE..

[19] Sanna D (2014). New mitochondrial and nuclear primers for the Mediterranean marine bivalve *Pinna nobilis*. Medit. Mar. Sci..

[20] González-Wangüemert (2015). Highly polymorphic microsatellite markers for the Mediterranean endemic fan mussel. Pinna nobilis. Medit. Mar. Sci..

[21] Selkoe KA, Toonen RJ (2006). Microsatellites for ecologists: a practical guide to using and evaluating microsatellite markers. Ecol. Lett..

[22] Marchesiello P, McWilliams JC, Shchepetkin A (2001). Open boundary conditions for long-term integration of regional oceanic models. Ocean Modell..

[23] Smith WHF, Sandwell DT (1997). Global sea floor topography from satellite altimetry and ship depth soundings. Science..

[24] Lellouche JM (2013). Evaluation of global monitoring and forecasting systems at Mercator Océan. Ocean Sci..

[25] Skamarock, W. C., *et al**A description of the advanced research WRF version 2* (No. NCAR/TN-468 + STR). National Center For Atmospheric Research Boulder Co Mesoscale and Microscale Meteorology Div (2005).

[26] Fairall CW, Bradley EF, Hare JE, Grachev AA, Edson J (2003). Bulk parameterization of air-sea fluxes: Updates and verification for the COARE algorithm. J. Climate..

[27] Sayol JM (2013). Sea surface transport in the Western Mediterranean Sea: A Lagrangian perspective. J. Geophys. Res. Oceans..

[28] Cabanellas-Reboredo M, Alvarez E (2009). Recruitment of *Pinna nobilis* (Mollusca: Bivalvia) on artificial structures. Mar. Biodivers. Rec..

[29] Sambrook, E., Fritsch, F., Maniatis, T. Molecular cloning. Cold Spring Harbour Press, New York (1989).

[30] Folmer O, Black M, Hoeh W, Lutz R, Vrijenhoek R (1994). DNA primers for amplification of mitochondrial cytochrome c oxidase subunit I form diverse metazoan invertebrates. Mol. Mar. Biol. Biotechnol..

[31] Toonen RJ, Hughes S (2001). Increased throughput for fragment analysis on an ABI PRISM (R) automated sequencer using a membrane comb and STRand software. J. BioTechnol..

[32] Hall, T. *BIOEDIT*. North Carolina State University, Raleigh, North Carolina (2001).

[33] Excoffier L, Laval G, Schneider S (2005). Arlequin ver. 3.0: An integrated software package for population genetics data analysis. Evol. Bioinform..

[34] Tajima F (1989). Statistical method for testing the neutral mutation hypothesis by DNA polymorphism. Genetics..

[35] Fu YX (1997). Statistical test of neutrality of mutations against population growth, hitchhiking and background selection. Genetics..

[36] Rogers AR, Harpending H (1992). Population growth makes waves in the distribution of pairwise genetic differences. Mol. Biol. Evol..

[37] Ramos-Onsins SE, Rozas J (2002). Statistical properties of new neutrality test against population growth. Mol. Biol. Evol..

[38] Librado P, Rozas J (2009). DnaSP version 5: a software for comprehensive analysis for DNA polymorphism data. Bioinformatics..

[39] Clement M, Posada D, Crandall KA (2000). TCS: a computer program to estimate gene genealogies. Mol. Ecol..

[40] Belkhir, K., Borsa, P., Goudet, J., Chicki, L. & Bonhomme, F. GENETIX 4.05, logiciel sous WindowsTM pour la génetique des populations, http://www.univmontp2.fr/~genetix (1999).

[41] Raymond, M. & Rousset, F. (Genepop (Version-1.2):Population genetics software for exact test and ecumenicism. *J. Hered*. **86**, 248–249 (1995).

[42] Van Oosterhout C, Hutchinson WF, Wills DPM, Shipley P (2004). MicroChecker: software for identifying and correcting genotyping errors in microsatellite data. Mol. Ecol. Notes..

[43] Chapuis MP, Estoup A (2007). Microsatellite null alleles and estimation of population differentiation. Mol. Biol. Evol..

[44] Weir BS, Cockerham CC (1984). Estimating F-Statistics for the Analysis of Population Structure. Evolution..

[45] Gerlach G, Jueterbock A, Kraemer P, Deppermann J, Harmand P (2010). Calculations of population differentiation based on Gst and D: forget Gst but not all statistics. Mol. Ecol..

[46] Jost L (2008). GST and its relatives do not measure differentiation. Mol. Ecol..

[47] Keenan K, McGinnity P, Cross TF, Crozier WW, Prodöhl P (2013). A. diveRsity: An R package for the estimation and exploration of population genetics parameters and their associated errors. Methods Ecol. Evol..

[48] R Core Team R: A language and environment for statistical computing. R Foundation for Statistical Computing, Vienna, Austria. http://www.R-project.org/ (2013).

[49] Pritchard JK, Stephens M, Donnelly P (2000). Inference of population structure using multilocus genotype data. Genetics.

[50] Evanno G, Regnaut S, Goudet J (2005). Detecting the number of clusters of individuals using the software Structure: a simulation study. Mol. Ecol..

[51] Earl DA, von Holdt BM (2012). Structure Harvester: A website and program for visualizing Structure output and implementing the Evanno method. Conserv. Genet. Resour..

[52] Piry S (2004). GeneClass2: A Software for Genetic Assignment and First-Generation Migrant Detection. J. Hered..

[53] Rannala B, Mountain JL (1997). Detecting immigration by using multilocus genotypes. P. Natl. Acad. Sci. USA.

[54] Paetkau D, Slade R, Burden M, Estoup A (2004). Direct, real-time estimation of migration rate using assignment methods: a simulation-based exploration of accuracy and power. Mol. Ecol..

[55] Beerli P, Felsenstein J (2001). Maximum likelihood estimation of a migration matrix and effective population sizes in n subpopulations by using a coalescent approach. P. Natl. Acad. Sci. USA.

[56] Mantel N (1967). The detection of disease clustering and a generalized regression approach. Cancer Res..

[57] Berline L, Rammou A, Doglioli A, Molcard A, Petrenko A (2014). A connectivity-based Eco-Regionalization Method of the Mediterranean Sea. PLoS ONE..

[58] Posada D, Crandall KA (2001). Intraspecific gene genealogies: trees grafting into networks. Trends Ecol. Evol..

[59] Patarnello T, Volckaert FAMJ, Castilho R (2007). Pillars of Hercules: is the Atlantic–Mediterranean transition a phylogeographical break?. Mol. Ecol..

[60] Borrero-Pérez GH, González-Wangüemert M, Marcos C, Pérez-Ruzafa A (2011). Phylogeography of the Atlanto-Mediterranean sea cucumber *Holothuria mammata*: the combined effects of historical processes and current oceanographical pattern. Mol. Ecol..

[61] Arnaud-Haond (2007). Vicariance patterns in the Mediterranean Sea: east–west cleavage and low dispersal in the endemic seagrass Posidonia oceanica. J. Biogeogr..

[62] Albertom F. (2008). Genetic differentiation and secondary contact zone in he seagrass *Cymodocea nodosa* across the Mediterranean-Atlantic transition Region. J. Biogeogr..

[63] Lemer S, Buge B, Bemis A, Giribet G (2014). First molecular phylogeny of the circumtropical bivalve family Pinnidae (Mollusca, Bivalvia): Evidence for high levels of cryptic species diversity. Mol. Phylogenet. Evol..

[64] Shunter C (2011). Matching genetics with oceanography: directional gene flow in a Mediterranean fish species. Mol. Ecol..

[65] González-Wangüemert M, Cánovas F, Pérez-Ruzafa A, Marcos C, Alexandrino P (2010). Connectivity patterns inferred from the genetic structure of white seabream (*Diplodus sargus L*.). J. Exp. Mar. Biol. Ecol..

[66] Robinson, A.R., Leslie, W.G., Theocharis, A. & Lascaratos, A. Mediterranean Sea Circulation. Encyclopedia of Ocean Sciences. Academic press, 1689–1706 (2001).

[67] Galarza JA (2009). The influence of oceanographic fronts and early-life-history traits on connectivity among littoral fish species. P. Natl. Acad. Sci. USA.

[68] Prado P, Caiola N, Ibáñez C (2014). Habitat use by a large population of *Pinna nobilis* in shallow waters. Sci. Mar..

[69] Vicente N (1990). Estudio ecológico y protecció del molusco lamelibranquio *Pinna nobilis* L. 1758 en la costa mediterránea. Iberus..

[70] Ibáñez, C. Pla XXI. Directrius per a la conservació i dessenvolupament sostenible al delta de l’Ebre. (Plan XXI: Guideliness for conservation and sustainable development in the Ebro Delta). Sociedad Española de Ornitología/Birdlife. Tarragona, Spain (1997).

[71] Wright S (1969). Evolution and the genetics of populations. The theory of gene frequencies. University of Chicago Press.

[72] Vázquez-Luis, M. *et al*. S.O.S. *Pinna nobilis*: A Mass Mortality Event in Western Mediterranean Sea. *Frontiers in Marine Science* **4** (2017)

