# Supplementary material for: Genetic and oceanographic tools reveal high population connectivity and diversity in the endangered pen shell Pinna nobilis
Source: Sci Rep. 2018 Mar 19;8:4770. doi: 10.1038/s41598-018-23004-2 (PMC5859023; doi:10.1038/s41598-018-23004-2)
Supplement: Supplementary file 1 — Supplementary Tables [file 41598_2018_23004_MOESM1_ESM.pdf]

Supplementary Tables for:

Genetic and oceanographic tools reveal high population connectivity and diversity in the endangered pen shell *Pinna nobilis*

Marlene Wesselmann[1,2], Mercedes González-Wangüemert[1], Ester A. Serrão[1], Aschwin H. Engelen[1], Lionel Renault[3], José R. García-March[4], Carlos M. Duarte[5], and Iris E. Hendriks[2,6*]

[1]*CCMAR, Universidade do Algarve, Gambelas, 8005-139 Faro, Portugal.*

[2]*Global Change Department, IMEDEA, Instituto Mediterráneo de Estudios Avanzados, C/ Miquel Marqués 21,07190 Esporles, Spain.*

[3]*Department of Atmospheric and Oceanic Sciences, University of California, Los Angeles, California, USA*

[4]*Instituto de Investigación en Medio ambiente y ciencia Marina (IMEDMAR-UCV), Universidad Católica de Valencia, C/Explanada del Puerto s/n, Calpe (Alicante).*

[5]*King Abdullah University of Science and Technology (KAUST), Red Sea Research Center, Thuwal 23955-6900, Saudia Arabia*

[6]*Biology department, University of the Balearic Islands (UIB), crta. Valldemossa km 7,5, 07122 Palma de Mallorca*

*corresponding author: iris@imedea.uib-csic.es, tel. +34 971611359

Table S1. Sampling location and size for 24 populations of *Pinna nobilis* considered for the COI (243bp) analysis. Locations whose sequences were taken from Genbank: *1 Sanna et al ., 2013 (18), *2 Rabaoui et al., 2010 (17), *3 Katsares et al., 2009 (16).

	Location	Label	N
**-Western Mediterranean Mainland-**	Banyuls	BY	9
	The Ebro Delta	DE	9
	Alicante	AT	10
	Murcia	MU	9
**-Balearic Islands-**	Ibiza	IB	10
	Mallorca	MA	10
**-Elba Island-***1	Capo Enfola	EL	10
**-Corsica Island-***1	Cala Pesciu cane	CP	12
	Isola Piana	IP	13
**-Sardinia Island-***1	Baia di Porto Conte	BP	18
	Ospedale Marino	OS	21
	Molara	MO	11
	Capo Ceraso	CC	13
	Origina di Siracusa	OR	10
	Isola di la Madalena	MD	18
**-Sicily Island-***1	Mondello	MN	11
	Milazzo	ML	10
	Origina di Siracussa	OG	15
**-The Venetian Lagoon-***1	Ottagono Alberoni	VE	20
**-Tunisian Coast-***2	Monastir	M	9
	El Bibane	B	9
	El Ketef	K	17
**-Aegean Sea-***3	Aggeloyesori	AG	9
	Epanomi	EP	9

Table S2: Genetic differentiation (F_{ST}), median oceanographic distance (km) and median oceanographic transport time (days) for each pair of population of *Pinna nobilis* sampled in six localities in the Western Mediterranean: Banyuls (BY), the Ebro Delta (DE), Alicante (AT), Murcia (MU), Ibiza (IB), Mallorca (MA).

Pair of population	Genetic differentiation (F_{ST})	Median oceanographic distance (km)	Median oceanographic time (days)	Minimum oceanographic time (days)
MU - AT	-0.0059	757 ±135	19 ±15	4
DE – IBZ	-0.0037	418 ±107	33 ±9	20
MU – IB	-0.0036	718 ±259	31 ±9	19
IB – MA	-0.0016	293 ±89	25 ±21	6
AT - DE	0.0010	926 ±380	27 ±7	16
AT – MA	0.0022	886 ±118	36 ±5	23
DE – MU	0.0023	1859 ±118	59 ±13	35
DE – MA	0.0024	568 ±165	37 15	20
AT – IB	0.0056	364 ±105	35 ±10	8
BY – AT	0.0088	≥1859	≥59	≥35
MU – MA	0.0120	≥1859	≥59	≥35
BY – DE	0.0120	≥1859	≥59	≥35
BY – MA	0.0166	≥1859	≥59	≥35
BY – IB	0.0167	≥1859	≥59	≥35
BY – MU	0.0180	≥1859	≥59	≥35

Table S3: Genetic diversity estimated using 243 bp COI sequences, for *Pinna nobilis* sampled in 24 Mediterranean populations (N: number of individuals, Hap: number of haplotypes and exclusive haplotypes in brackets, H: haplotype diversity, Π: nucleotide diversity). Banyuls (BY), the Ebro Delta (DE), Alicante (AT), Murcia (MU), Ibiza (IB), Mallorca (MA) Elba (EL), Cala Pesciu Cane (CP) and Isola Piana (IP) in Corsica, Baia di Porto Conte (BP), Ospedale Marino (OS), Molara (MO), Capo Ceraso (CC), Oristano (OR) and Isola di La Maddalena (MD) in Sardinia, Mondello (MN), Milazzo (ML) and Origina di Siracusa (OR) in Sicily, the Venetian Lagoon (VE), Monastir (M), El Bibane (B) and El Ketef (K) at the Tunisian Coast, and in the Aegean Sea: Epanomoi (EP) and Aggeloyesori (AG).

Population	N	Hap	Polymorphic sites	H	Π
BY	9	4(0)	3	0.7500	0.0038
DE	9	3(2)	2	0.4167	0.0018
AT	10	7(2)	9	0.8667	0.0099
MU	9	1(0)	0	0.0000	0.0000
IB	10	4(1)	3	0.5333	0.0024
MA	10	4(2)	4	0.5333	0.0032
EL	10	4(2)	5	0.8000	0.0082
CP	12	5(1)	5	0.5758	0.0039
IP	13	9(0)	9	0.9487	0.0091
MO	11	5(0)	5	0.7636	0.0067
OS	21	6(0)	6	0.6857	0.0038
BP	18	5(1)	6	0.7250	0.0030
CC	13	3(0)	2	0.7050	0.0020
OR	10	6(0)	4	0.9110	0.0050
MD	18	7(0)	6	0.8950	0.0040

ML	10	5(2)	5		0.8667	0.0081
MN	11	6(2)	4		0.8364	0.0051
OG	15	7(2)	4		0.8860	0,0050
B	9	2(0)	1		0.5560	0.0009
K	17	1(0)	0		0.3820	0.0000
M	9	3(0)	2		0.6940	0.0025
VE	20	10(4)	8		0.8947	0.0074
AG	9	2(0)	1		0.3889	0.0016
EP	9	4(0)	4		0.6944	0.0050

Table S4: Population pairwise F_{ST} estimated for sequences of COI, for *P. nobilis* sampled in 24 localities in the Mediterranean Banyuls (BY), the Ebro Delta (DE), Alicante (AT), Murcia (MU), Ibiza (IB), Mallorca (MA) Elba (EL), Cala Pesciu Cane (CP) and Isola Piana (IP) in Corsica, Baia di Porto Conte (BP), Ospedale Marino (OS), Molara (MO), Capo Ceraso (CC), Oristano (OR) and Isola di La Maddalena (MD) in Sardinia, Mondello (MN), Milazzo (ML) and Origina di Siracusa (OR) in Sicily, the Venetian Lagoon (VE), Monastir (M), El Bibane (B) and El Ketef (K) at the Tunisian Coast, and in the Aegean Sea: Epanomoi (EP) and Aggeloyesori (AG). F_{ST} values in grey are significant after Bonferroni correction ($p<0.0002$).

	BY	DE	AT	MU	IB	MA	EL	IP	CP	MO	OS	BP	CC	OR	MD	ML	MN	OG	B	K	M	VE	AG	EP
**BY**	0																							
**DE**	0,092	0																						
**AT**	0,075	0,028	0																					
**MU**	0,150	0	0,055	0																				
**IB**	0,100	-0,001	0,018	-0,011	0																			
**MA**	0,088	-0,003	0,029	-0,011	-0,029	0																		
**EL**	0,200	0,186	-0,014	0,213	0,115	0,135	0																	
**IP**	0,067	0,052	-0,031	0,053	0,026	0,035	-0,004	0																
**CP**	0,092	0,009	-0,020	0,001	-0,038	-0,022	0,043	-0,017	0															
**MO**	0,248	0,240	0,018	0,275	0,214	0,031	0,039	0,013	0,142	0														
**OS**	0,162	0,094	0,033	0,092	0,022	0,035	0,072	0,049	-0,020	0,246	0													
**BP**	0,103	-0,004	0,028	-0,030	-0,036	-0,019	0,127	0,025	-0,037	0,207	0,023	0												
**CC**	0,155	0,068	0,030	0,081	-0,012	-0,007	0,128	0,040	-0,048	0,276	-0,037	-0,013	0											
**OR**	0,135	0,089	0,030	0,112	0,030	0,027	0,063	0,052	0,005	0,238	-0,003	0,040	-0,003	0										
**MD**	0,103	0,026	0,007	0,022	-0,016	-0,004	0,066	0,024	-0,043	0,198	-0,018	-0,010	-0,040	-0,036	0									
**ML**	0,206	0,193	0,009	0,222	0,117	0,131	-0,086	0,028	0,051	0,129	0,047	0,134	0,105	0,012	0,044	0								
**MN**	0,192	0,169	0,016	0,203	0,117	0,086	0,002	0,036	0,002	0,222	-0,026	0,087	0,012	0,005	0,012	-0,031	0							
**OG**	0,170	0,127	0,060	0,140	0,068	0,073	0,045	0,079	0,006	0,247	0,032	0,082	0,043	0,018	0,033	0,011	-0,028	0						
**B**	0,125	0,000	0,022	0,000	-0,061	-0,050	0,147	0,030	-0,052	0,256	0,003	-0,060	-0,047	0,021	-0,036	0,141	0,090	0,066	0					
**K**	0,234	0,060	0,121	0,000	0,042	0,042	0,299	0,110	0,052	0,360	0,140	0,005	0,142	0,187	0,066	0,308	0,238	0,202	0,060	0				
**M**	0,125	0,050	-0,017	0,083	-0,028	0,006	0,052	-0,014	-0,046	0,131	0,024	-0,028	0,027	0,047	-0,014	0,075	0,049	0,063	0,007	0,158	0			
**VE**	0,338	0,331	0,178	-0,348	0,271	0,271	0,079	0,206	0,214	0,338	0,174	0,291	0,229	0,117	0,190	-0,001	0,071	0,121	0,276	0,41	0,254	0		
**AG**	0,142	0,062	0,066	0,125	0,050	0,040	0,204	0,068	0,045	0,258	0,122	0,035	0,117	0,118	0,063	0,211	0,194	0,150	0,083	0,206	0,100	0,342	0	
**EP**	0,113	0,062	-0,022	0,083	0,016	0,073	0,023	-0,010	-0,035	0,127	-0,004	0,001	0,004	0,009	-0,015	0,030	0,030	0,051	0,008	0,158	0,035	0,187	0,093	0

Table S5: Population pairwise F_{ST} values estimated for sequences of COI (590 bp), for *Pinna nobilis* sampled in six localities in the Western Mediterranean: Banyuls (BY), the Ebro Delta (DE), Alicante (AT), Murcia (MU), Ibiza (IB), Mallorca (MA).

Locations	BY	DE	AT	MU	IB	MA
Banyuls	-					
Ebro Delta	0.1000	-				
Alicante	0.0898	0.0455	-			
Murcia	0.1607	0.1250	0.0914	-		
Ibiza	0.0902	-0.0023	0.0391	-0.0951	-	
Mallorca	0.0677	0.0272	0.0540	0.1120	0.0033	-

Table S6: Neutrality tests and demographic expansion parameters for *Pinna nobilis* populations in the Western Mediterranean Sea: Banyuls, the Ebro Delta, Alicante , Murcia, Ibiza, Mallorca. Tajima's D and R2 values with * are significant at *P*<0.05 and Fu's Fs values with ** are significant at *P*<0.02

Population	Tajima's D	Fu's Fs	R2
Banyuls	-0.3983	-1.2600	0.1374
Ebro Delta	-1.3624*	-1.0811**	0.2079
Alicante	-0.8975	-1.4938	0.1085*
Murcia	0.9862	0.8495	0.2500
Ibiza	-1.7411*	-3.8769**	0.1000*
Mallorca	-2.2630*	-3.4235**	0.0308*

Table S7: Neutrality tests and demographic expansion parameters for *Pinna nobilis* populations in the Mediterranean Sea: Banyuls (BY), the Ebro Delta (DE), Alicante (AT), Murcia (MU), Ibiza (IB), Mallorca (MA) Elba Island (EL), Cala Pesciu Cane (CP) and Isola Piana (IP) in Corsica, Ospedale Marino (OS) and Molara (MO) in Sardinia, Mondello (MN) and Milazzo (ML) in Sicily and the Venetian Lagoon (VE), Tunisian Coast (TU), and in the Aegean Sea: Epanomoi (EP) and Aggeloyesori (AG). Tajima's D and R2 values with * are significant at $P<0.05$ and Fu's Fs values with ** are significant at $P<0.02$

Population	Tajima's D	Fu's Fs	R2
BY	-0.5515	-1.1568	0.1617
DE	-1.3624*	-1.0811*	0.2079
AT	-1.04488	-2.6712	0.1086*
MU	0.0000	0.0000	0.0000
IB	-1.5622*	-1.9637**	0.1528
MA	-1.6670*	-1.3446**	0.1658
EL	0.5273	0.5924	0.2280
CP	-1.5273*	-1.9778	0.1098
IP	-0.9093	-4.7696**	0.1043
MO	-0.1587	-0.8647	0.1569
OS	-1.3652	-2.3609	0.1134
BP	-1.8490*	-1.9550	0.1230
CC	-0.4620	0.4130	0.1643
OR	-0.520	-3.237**	0.1434
MD	-1.3680	-3.380**	0.1009
ML	-0.2979	-2.8715**	0.1435
MN	-0.6564	-5.0997**	0.1003
OG	0.1190	-3.3800**	0.1555
B	-1.088	-0.2630**	0.3143
K	0.0000	0.0000	0.0000
M	-0.5830	-0.5320	0.1848
VE	0.4772	-0.6276**	0.1876
AG	0.15647	0.4774	0.1944*
EP	-0.6891	-0.6273	0.1848

Table S8. F_{ST} estimates with microsatellite loci and 95% CI among adults of *Pinna nobilis* from 6 Western Mediterranean localities: Banyuls (BY), the Ebro Delta (DE), Alicante (AT), Murcia (MU), Ibiza (IB), Mallorca (MA) without ENA correction and with correction.

	Without ENA correction		With ENA correction	
	FST	95% CI	FST	95% CI
BY-DE	0.0120	(0.0034, 0.0209)	0.0101	(0.0024, 0.0183)
BY-AT	0.0088	(-0.0051, 0.0233)	0.0094	(-0.0033, 0.0225)
BY-MU	0.0180	(-0.0030, 0.0427)	0.0141	(-0.0009, 0.0299)
BY-IB	0.0167	(0.0026, 0.0315)	0.0200	(0.0066, 0.0352)
BY-MA	0.0166	(0.0017, 0.0335)	0.0177	(0.0002, 0.0374)
DE-AT	0.0010	(-0.0057, 0.0081)	0.0032	(-0.0030, 0.0095)
DE-MU	0.0023	(-0.0068, 0.0113)	0.0038	(-0.0042, 0.0119)
DE-IB	-0.0037	(-0.0138, 0.0065)	0.0017	(-0.0066, 0.0101)
DE-MA	0.0024	(-0.0055, 0.0121)	0.0053	(-0.0031, 0.0148)
AT-MU	-0.0059	(-0.0143, 0.0054)	-0.0052	(-0.0119. 0.0021)
AT-IB	0.0056	(-0.0054, 0.0178)	0.0070	(-0.0030, 0.0182)
AT-MA	0.0022	(-0.0075, 0.0119)	0.0034	(-0.0043, 0.0108)
MU-IB	-0.0036	(-0.0168, 0.0112)	0.0018	(-0.0102, 0.0154)
MU-MA	0.0120	(0.0009, 0.0256)	0.0092	(0.0014, 0.0171)
IB-MA	-0.0016	(-0.0099, 0.0081)	-0.0011	(-0.0066, 0.0043)

Table S9. Pairwise F_{ST} among population pairs of *Pinna nobilis* juveniles from the Western Mediterranean estimated with microsatellites. Banyuls (BY) and Mallorca (MA). No significant differentiation was detected with $F_{ST.}$

Locations	BY (adult)	BY (juvenile)	MA (adult)	MA (juvenile)
BY (adult)	-			
BY (juveniles)	0.0131	-		
MA (adult)	0.0356	-0.0005	-	
MA (juvenile)	0.0286	-0.0003	0.0091	-

Table S10: Migration rates (M = m/μ) calculated with Migrate-n based on 10 microsatellite loci of the sampled *P. nobilis* populations from the Western Mediterranean: Banyuls (BY), the Ebro Delta (DE), Alicante (AT), Murcia (MU), Ibiza (IB), Mallorca (MA). Three different analyses were run; corresponding to the genetically differentiated clusters identified by STRUCTURE.

Migration rates ($M = m/\mu$)					
Analysis 1:BY-DE-AT		Analysis 2:AT-MU-IB		Analysis 3: DE-IB-MA	
$M_{BY \rightarrow DE}$	9.050	$M_{AT \rightarrow MU}$	2.517	$M_{DE \rightarrow IB}$	6.767
$M_{BY \rightarrow AT}$	1.050	$M_{AT \rightarrow IB}$	4.750	$M_{DE \rightarrow MA}$	4.900
$M_{DE \rightarrow BY}$	0.650	$M_{MU \rightarrow AT}$	9.4167	$M_{IB \rightarrow DE}$	3.500
$M_{DE \rightarrow AT}$	2.067	$M_{MU \rightarrow IB}$	4.617	$M_{IB \rightarrow MA}$	5.367
$M_{AT \rightarrow BY}$	0.417	$M_{IB \rightarrow AT}$	4.683	$M_{MA \rightarrow DE}$	4.500
$M_{AT \rightarrow DE}$	8.383	$M_{IB \rightarrow MU}$	2.950	$M_{MA \rightarrow IB}$	2.433

Table S11: Number of juveniles assigned (*N=70*) to one of the 6 adult populations; Banyuls (BY), the Ebro Delta (DE), Alicante (AT), Murcia (MU), Ibiza (IB), Mallorca (MA). Juveniles with a probability <0.05 of belonging only to a single adult population, were assigned to that population. "Unassigned" juveniles had probabilities ≥0.05 of belonging to more than one source. Juveniles with probability of origin <0.05 for *all* adult populations were considered to have originated from an unsampled so

Juveniles	Assigned adult population						unassigned	unsampled source
	BY	DE	AT	MU	IB	MA		
BY (35)	1	0	0	0	0	0	5	29
MA (35)	0	1	3	0	0	2	2	26
